# Perceptions and Experiences of Caregivers on Child Injuries: A Qualitative Study from Central India

**DOI:** 10.1007/s10935-022-00682-3

**Published:** 2022-05-27

**Authors:** Ashish Pathak, Akindayo Ogunbayo, Tanwi Trushna, Shweta Khare, Aditya Mathur, Salla Atkins, Vishal Diwan

**Affiliations:** 1 Health Systems and Policy (HSP): Medicines, Focusing Antibiotics, Department of Global Public Health, Karolinska Institutet, Tomtebodavagen 18A, 171 77 Stockholm, Sweden; 2 Department of Pediatrics, Ruxmaniben Deepchand Gardi Medical College, Ujjain, Madhya Pradesh 456006 India; 3 Global Health and Development, Faculty of Social Sciences, Tampere University, Arvo Ylpön katu 34, 33520 Tampere, Finland; 4 ICMR-National Institute for Research in Environmental Health, Bhopal, India; 5 Department of Public Health and Environment, Ruxmaniben Deepchand Gardi Medical College, Ujjain, Madhya Pradesh 456006 India; 6 Social Medicine, Infectious Diseases and Migration (SIM), Department of Global Public Health, Karolinska Institutet, Tomtebodavagen 18A, 171 77 Solna, Sweden

**Keywords:** Qualitative study, Caregivers, Perceptions, Child injury, India, Falls, Burns, Drowning, Suffocation

## Abstract

To explore caregivers’ perceptions of childhood injuries in the rural and urban areas of India, with a focus on causes, consequences, prevention, and treatment. We conducted eight focus group discussions with fifty female caregivers in rural and urban areas of Ujjain in Central India and used thematic content analysis. The caregivers identified how children injured themselves through falls, road traffic injuries, metallic nails and tool injuries, ingestions of foreign objects and poisons, burns, drowning, and suffocation. The reported consequences of injuries ranged from pain, infections, scar formation, phobia, stigma, and emotional stress to complications like physical disability, loss of eyesight, head injury, paralysis, and even death. Many caregivers blamed children and their mischievousness for the injuries and failed to realise/acknowledge the role of better supervision and environmental modifications in injury prevention. Caregivers used several first aid methods to respond to injuries. These included applying pressure to stop bleeding during fall and road traffic injuries, inducing vomiting by giving the poison victims saltwater to drink, and tobacco leaves to chew. In addition, some caregivers resorted to using coconut oil and toothpaste on burnt skin and giving back blows for choking. Caregivers in communities had experiences of different types of child injuries. Further education on need for better supervision, relevant environmental modification and appropriate first aid treatment of various injuries is required.

**Supplementary Information:**

The online version contains supplementary material available at 10.1007/s10935-022-00682-3.

## Introduction

Child injury is one of the significant causes of child death globally. In 2008, about 950,000 children under 18 years died due to injuries (Peden et al., 2008). Approximately 90% of child injuries are unintentional and include injury categories such as road traffic injuries (22.3%), drowning (16.8%), poisoning (3.9%), fire-related burns (9.1%), and falls (4.2%) (Peden et al., 2008). In total, 31.1% of injuries are categorized into "other unintentional child injuries”, which includes smothering, asphyxiation, choking, animal and venomous bites, hypothermia, and hyperthermia, as well as natural disasters (Peden et al., 2008). The remaining injuries are intentional, including homicide (5.8%), self-inflicted injuries (4.4%), and war (2.3%) (Peden et al., 2008).

Child injuries are associated with a child’s developmental milestones (Flavin et al., 2006). For example, children aged 1 to 3 years are in the oral phase of development and tend to place multiple objects into the mouth, increasing the risk of accidental ingestion of poison (Flavin et al., 2006). In addition, as children grow, they explore their surrounding environments with curiosity and start actively running and playing in their neighbourhood without possessing the requisite skills to identify and deal with potential health hazards. Thus the physical environment may start playing an increasing role in child injuries (Garzon, 2005; Schwebel et al., 2009). Therefore, to prevent injuries community-based interventions that include increased supervision through parental education or modifying or changing the overall design of the physical environment have been suggested (Peden et al., 2008; Towner & Ward, 1998).

The caregivers’ role in child injuries cannot be overemphasized. The likelihood of unintentional injury-related death in children is threefold higher among unsupervised children when compared to supervised children (Khatlani et al., 2017). Some unintentional injuries may result from inadequate supervision or a caregiver’s failure to protect the child from hazards causing injury that may constitute child neglect (Schnitzer et al., 2011). In contrast, others may be due to oversight and lack of knowledge about developmental periods or a child's typical behaviour at a certain age (Peden et al., 2008). The caregivers’ perceptions of child injury are critical because they can help to identify risks and prevent injuries (Huynh et al., 2017). Also, caregivers’ previous experiences can significantly assist in putting in place measures to make their immediate environment safe for children to avoid injuries. In designing appropriate child injury prevention interventions, local knowledge of the setting is invaluable, and thus exploring caregivers’ perceptions and experiences can provide helpful information.

However, in India, the study of child injuries has been focused solely on quantitative cross-sectional measurements using epidemiological data (Inbaraj et al., 2017; Sharma et al., 2018). There is a paucity of published literature that explores the caregiver perceptions of child injuries and their prevention in the Indian setting. Given the value of caregivers’ perceptions and experiences of child injuries and their prevention in designing appropriate intervention strategies, this qualitative study was conceptualized to explore how caregivers perceived child injuries and their prevention and to gain insight from their experiences.

## Methods

### Study Setting

This study was conducted in the Ujjain district of Madhya Pradesh state, India. Ujjain is one of the 52 administrative districts of Madhya Pradesh (2020), with a population of more than 72 million people (Madhya Pradesh Population, 2020). Approximately 7.8% of Ujjain’s inhabitants live below the poverty line (Bhanumurthy et al., 2016), which is lower than the rate of the Indian average (21.9%) (Ministry of statistics & programme implementation, 2018). The average literacy rate is 72.34%, with female literacy (60.74%) lagging behind male literacy (83.46%) (Ujjain District Population Census, 2020). The maternal mortality ratio in the district is 176 per 100,000 live births; the infant mortality rate is 54 per 1000 live birth, and the under-five mortality rate is 70 per 1000 live births (Annual Health Survey, 2020), all of which are higher than the national average (Maternal & Adolescent Healthcare, 2020; Goal 3: Ensure healthy lives and promote well-being for all at all ages, 2020).

### Research Team

A multidisciplinary team of researchers with public health backgrounds conducted this study. VD is a qualitative researcher and public health specialist. AP is a paediatrician and health system researcher. SK is a PhD student working in qualitative and quantitative research in the selected study area. AM is a public health researcher and a qualified practitioner of the Indian system of medicine. SA is a trained qualitative researcher and Social Scientist, and TT is a medical doctor trained in qualitative research methods. The team consist of four Male (VD, AP, AO, AM) and three Female members (SA, TT and SK). AP and VD initiated the concept and developed the topic guide in collaboration with SA, AM and SK. AM, SK and VD conducted pilot-testing of the topic guide. SK along with two more female research assistants conducted the FGDs. The transcription and translation was done by VD and AM. AO, SA, VD, AP and TT analyzed data. AO and SA developed initial draft. TT and VD revised the draft. All other authors contributed in reviewing and editing.

### Participants, Sampling, and Data Collection

We used purposive sampling for the selection of sites and participants. We purposively decided to conduct the current study in the rural and urban field practice areas of R. D. Gardi Medical College, located in Ujjain. The medical college provides preventive, curative, and health-promoting services to these areas and has built a rapport with the community. From all the villages and urban sites covered by the medical college, we randomly selected 5 villages and 2 urban sites (total 7 sites) to conduct focus group discussions (FGDs). With the help of Anganwadi (a type of childcare centre in India) from each rural and urban site, we compiled the list of eligible households (i.e. having at least one member under 18 years of age) in each site. Using these lists as our sampling frame, we further randomly selected 105 households (15 from each of the seven areas). Members of the research team, including female staff, accompanied by an Anganwadi worker, visited these households to inform them about the study and the date/time of the interview. Since child caregivers were female in all visited homes, no male caregiver could participate. Instead, the primary female caregiver in each household was invited to participate. For the convenience of consenting families, FGDs were held either in one of the participants' homes or nearby schools. We ascertained that each focus group consisted of 6 to 8 caregivers (mothers and grandmothers). At the onset, details such as age, education, and occupation of the participants was collected.

Trained female FGD facilitators used an open-ended interview guide to explore participants’ perceptions, experiences of common causes, consequences, treatments, and prevention of childhood injuries (see Appendix 1 of Supplementary Material). The topic guide was developed through consultation with subject experts and local health workers and piloted to assess any ambiguity in rural and urban sites similar to the study area. However, child caregivers could not be directly involved in developing this topic guide. The FGDs were held in Hindi, the local language. The duration of the FGDs ranged from 40 to 55 min. All discussions were audio-recorded and transcribed verbatim at the end of the interview sessions. The transcripts were translated from the Hindi language to English by the researchers in India.

### Data Analysis

We used thematic content analysis and a deductive approach to analyze the data, following guidance from Krippendorf (2004), Graneheim and Lundman (2004). First, we read the transcripts many times to get familiarized with the data. Next, the texts were condensed, aggregated, and grouped under the same heading while the core contents of the text were preserved (Graneheim & Lundman, 2004). Finally, the aggregated and grouped texts were abstracted to create codes, categories, and themes.

Trustworthiness was achieved by thoroughly reporting the methodology (Elo et al., 2014) and including quotes from the discussions in the paper. During data analysis, special attention was given to ensure that the opinions of the participants were accurately represented based on the provided data (Supplementary Table 1 of Supplementary Material).

### Ethical Statement

The Institutional Ethical Committee of RD Gardi Medical College approved the study (No 354-08-01-2014). The research team briefed and obtained informed consent from all participants before the FGD. The researchers maintained the confidentiality of the participants.

## Results

Of the 105 families approached, 67 consented for participation during the first point of contact. A total of 8 FGDs were conducted with 50 participants who arrived for the FGDs on the pre-determined date and venue (Table 1). Thus, a total of 23 rural and 27 urban female caregivers ranging from 19 to 53 years in age with a mean 4.6 ± 2.8 years of schooling participated in the FGDs. 62% of the participants were housewives and 26% were involved in manual labour related work.

**Table 1:** Description of FGDs participants

FGD	Location	Number of participants	Age range (Years)	Number of years of schooling (Mean Years ± *SD*)	Occupation profile
1	Rural	6	19–32	4.2 ± 2.4	HW = 4, L = 2
2	Rural	7	21–31	3.4 ± 2.8	HW = 5, L = 1, O = 1
3	Urban	7	35–50	3.7 ± 2.9	HW = 4, L = 2, O = 1
4	Urban	6	19–30	3.2 ± 2.5	HW = 4, L = 2
5	Rural	5	35–50	3.6 ± 3.5	HW = 3, L = 2
6	Rural	5	23–29	5.4 ± 4.3	HW = 3, L = 1, O = 1
7	Urban	6	35–53	7.8 ± 1.7	HW = 3, L = 1, O = 2
8	Urban	8	21–32	6.3 ± 2.3	HW = 5, L = 2, O = 1
Total		50	19–53	4.6 ± 2.8	HW = 31, L = 13, O = 6

*HW* house wife, L = Manual Labour-related work, O = Other work

Following themes emerged: caregivers’ perceptions and experiences of common causes of child injuries, consequences of child injuries, caregivers' experience of first aid treatment during injury, and caregivers’ experiences of injury prevention. These are described in more detail below.

### Caregivers’ Perception and Experiences of Common Causes of Child Injuries

Caregivers identified how children commonly sustained injuries. These injuries fell into different categories of falls, road traffic injuries, metallic nails and tool injuries, ingestions of foreign objects and poisons, burns, drowning, and suffocation (Table 2).

**Table 2:** Caregivers’ perception on common injuries and knowledge of injury intervention

	Common injuries	Knowledge of injury intervention
Fall	Fall while learning to walk, while playing, from bed, from climbing staircase, while riding bicycle, during climbing, from colliding with moving objects	Application of pressure to stop bleeding. Massage. Hospitalisation
Road traffic Injury	Motorcycle accident, car accident, collision with pedestrians	Application of pressure to stop bleeding, taking the victim to hospital
Metallic nails and tool injuries	Stepping on nails, scissors, knives and blade injury	Taking the victim to hospital
Ingestion of foreign object/poisons	Ingestion of objects such as coins, pebbles, chalks, seeds and food. Ingestion of harmful substances such as kerosene, rat poison, pesticides and fertilizer	Induced vomiting by making the victim drink saltwater or chew tobacco leaf. Taking the victim to hospital
Injuries caused by animals	Dog bite, snake bite, scorpion stings, chase by dog, monkey and buffalo	Tying the region to prevent spread of the venom, spiritual cleansing
Burns	Hot water, hot tea, fall inside hot frying pan	Application of coconut oil, toothpaste. Hospitalisation
Drowning	Fall inside well, drowning during swimming	Taking off victim’s clothing, taking the victim to hospital
Suffocation	Suffocation due to object trapped in the respiratory tract, during pillow fight and during breast feeding	Back blow for suffocation due to choking

Several caregivers in various FGD attributed injuries to child-related factors, especially when discussing injuries due to falls, burns, ingestion of foreign objects and suffocation. One woman said:

*This boy is very mischievous. As he plays inside, he falls [demonstrates]. He is so naughty that, recently, he fell and hit his head on the gate. (FGD number 3)*

Some caregivers thought they had to supervise their children continuously to prevent them from injuring themselves.

Caregivers frequently mentioned falls in the FGDs. Almost all of the participants could recall two or more cases of fall-related injury. However, the caregivers considered falls inevitable among growing children, especially those that did not lead to serious injury. They explained that children encountered fall-related injuries while learning to walk, play, climb staircases, ride bicycles, climb heights, jump or sleep on the bed, and collide with moving objects such as vehicles and scooters. One participant explained how her daughter sustained injuries on two different occasions:

*While children were playing, they bumped into one another, and she fell, resulting in injuries. On another occasion, she was sitting, and another child pushed her. She fell off the bed leading to bone injury, and she could not raise her hand properly for eight days. (FGD number 4).*

Caregivers regarded falls in a child learning to walk as expected except in the case of a child who developed neurological sequelae due to falls while learning to walk. In many cases, falls on staircases led to orthopaedics injuries and required several days of hospitalization. Caregivers also recalled different fall events while children climbed trees and parked vehicles.

#### Road Traffic Injuries

The road traffic injuries mentioned in the groups included motorcycle accidents, car accidents, and vehicular collisions with pedestrians. Participants noted that children were victims of auto crash injuries as passengers commuting in motor vehicles. For example, a participant described a motorcycle accident occurring due to speeding and loss of control while the driver's wife and son were passengers. In this case, the child was severely injured and bled profusely, which led to several days of hospitalization. In addition to this case, there were several cases of children playing on the road, which led to accidents:

*The driver suddenly applied the brake, and he hit the boy. The vehicle rider was kind enough, felt regret and carried him to our door on the upper floor; otherwise, we would not have known of the incident. (FGD number 3)*

#### Metallic Objects and Tools Injuries

Caregivers reported that children were involved in many mischievous activities, which led to injuries of varying severity, especially while playing with sharp objects, including knives and scissors. Participants discussed cases of children putting metallic objects into electric sockets, which led to electric shock. Caregivers also reported metallic nail injuries which occurred when children stepped on nails while playing, leading to bleeding and infections on several occasions.

*It was the gallery, where children were playing. The little kids prepared dresses for dolls, cutting the clothes with scissors. We didn't know how the scissors pierced her eye, and the black lid of the eye was severely damaged, and she lost her vision. She was a young girl of age between 8 and 10 years old. (FGD number 3)*

#### Injuries Caused by Animals

Caregivers also reported injuries caused by different kinds of animals. For example, some described dog bites with visible scars and wounds caused by being chased by animals, such as dogs, monkeys, cows, and buffalo.

A dog bit my eldest daughter. Nine years have passed; still one can see the mark on her skin. (FGD number 6)

Injuries caused by snakebite were considered high risk in the studied communities. Snakes were reportedly found in many houses crawling out from the household plumbing materials such as wastewater outlets, drainage pipes, flat platforms, which on many occasions bit the children. In addition, many caregivers reported scorpion stings.

*The boy was sleeping alongside his other two brothers when a scorpion bit him, and he started crying out loud. Everybody woke up and turned on the light. Then, they saw the scorpion close to the wall. The child was taken to the hospital in the middle of the night and was hospitalized for three days. (FGD number 5)*

#### Burn Injuries

Participants discussed burn injuries of varying severity,including fatal ones. Children sustained burns by spillage of hot water, hot tea, boiling food, and accidental contact with hot frying pans. Caregivers described that they cooked on the floor because of the stove design, and they considered this a major risk factor for burn injuries.

*I was cooking, and he was sitting near the burner. He slipped and fell on the hot frying pan. He got burnt and started bleeding. We had to take him to the hospital for treatment. (FGD number 5)*

#### Ingesting Foreign Objects and Poisons

Caregivers reported that children accidentally swallowed foreign objects like coins and poisonous substances that looked like food, leading to various degrees of injuries. Furthermore, caregivers said that some poisons have a good smell and taste, which attracted children to eat them.

She ate fertilizer used in wheat thinking it is sugar; it is like sugar. We were all worried and confused about how much she had eaten. I was sitting inside. On our way back, suddenly, she started vomiting many times. She fainted while vomiting, and we had to rush her to the hospital. (FGD number 7)

#### Drowning

In some communities where the use of underground water storage tanks was common, caregivers reported drowning as a common injury encountered by many children. Children accidentally fell inside open water tanks and underground wells, leading to fatal and non-fatal drowning.

*When my son was in his childhood, he fell inside the water tank while running around and shouting lizard. He fell in the water tank, and if he had been left there for a second more, the scenario would have been different. (FGD number 4)*

The open drainage system also led to near-drowning cases among the children. Similarly, children and adults often engaged in recreational swimming in the nearby rivers, leading to drowning in another community.

#### Suffocation

Suffocation injuries were reported during everyday activities such as eating, while other children suffocated during unsafe play with polythene bags and pillow fights. Many of the suffocation incidents were accidental, with non-fatal and fatal consequences. Little children were chiefly involved in ingestion-related suffocation where the ingested materials accidentally got trapped in the respiratory tract, leading to airflow obstruction*.*

*There is a threat in eating bananas. A boy died at Subhash Nagar while eating a banana. He ate hastily, and it entered the respiratory tract. (FGD number 4)*

Apart from the accidental ingestion of foreign bodies leading to occlusion of the respiratory tract, another caregiver described an incidence of suffocation that occurred while a young inexperienced woman was breastfeeding her infant.

### Consequences of Child Injuries

The consequences reported by participants ranged from minor problems such as pain, infection, scar formation, phobia, stigma, and emotional stress to severe complications like physical disability, loss of eyesight, head injury, paralysis, and even death (see Table 3). For example, a caregiver described how a child developed fear of multiple everyday things after sustaining burn injuries.

*Since he was burnt, he developed a phobia. He is too much scared of being left behind in a closed room. He runs out there. He is scared when the fan is working. You have to put on the light. He is scared of television and high-pitched sound. (FGD number 8)*

**Table 3:** Reported consequences of child injuries

SN	Consequences of child injuries	Types of injury
1	Pain	Fall, road traffic accident, burns
2	Infection	Fall, burns
3	Scar formation	Fall, burns, road traffic accident
4	Physical disability	Fall, road traffic accident
5	Phobia	Dog bites
6	Loss of eyesight	Injuries by metallic objects
7	Head injury	Fall, road traffic accident
8	Paralysis	Fall
9	Stigma	Resulting from paralysis due to fall
10	Emotional stress	Suffered by the parents of injured children who became blind
11	Death	Fall, road traffic injuries, Injuries from metallic objects, burns, drowning and suffocation

While some injuries resulted in short-term consequences that although painful resolved without residual effect, others caused severe long-term complications that persisted until adulthood. A caregiver said:

*[Imagine] if a spectator can feel the pain, how much pain the injured fellow might be feeling. (FGD number 1)*

### Caregivers’ Perception of Injury Prevention

In terms of preventing injuries, caregivers resorted to restricting children by closing the doors that led to the staircase, kitchen, and other places where the likelihood of the child getting injured was high. In addition, to prevent accidental ingestion of poisons, caregivers reported keeping medications and other potential toxins out of reach of children, for example, by keeping them in a closed room or on high surfaces in the house.

They suggested that animals should not be left to roam freely, especially dogs and cattle, and that animals should be kept away from children. However, another caregiver suggested that keeping children indoors will prevent animal attacks because animals have equal rights to move around freely.

*We keep them at home, allowing them to watch television. (FGD number 4)*

Their suggestions on preventing drowning included covering underground wells and drainage and ensuring that lifeguards are always present at the rivers used for swimming. Similarly, caregivers suggested that parents of underage drivers have essential roles in preventing road traffic accidents as they should advise their children to be vigilant, attentive to speed limits and slow down when approaching junctions.

Finally, some caregivers suggested that sending children to school is a good way of preventing injuries because children will be under the supervision of competent and trained caregivers in school. They believed the school environment was safe, and some even suggested that children take extra classes, especially when the parents are not around, instead of being left unattended or with older siblings.

*We decided to admit the little kid at playschool to prevent them from going out and getting injured by animals. (FGD number 6)*

One caregiver even suggested that injury prevention should be taught at schools because children trust whatever their teachers say.

### Caregivers’ Experiences of First Aid Treatment in the Events of Injuries

During the discussions, caregivers shared their knowledge of first aid treatment of the injured children. These suggestions included both traditional interventions and professional first aid. The type of first aid given to the injured child depended on the type of injury the child had, the severity of the injury, and the caregivers’ knowledge of first aid.

According to participants, when children fell, leading to bleeding wounds, they applied pressure to stop the bleeding and cleaned the area with antiseptic liquid. Then, they took the victims to the hospital for treatment in complicated cases and asked for a tetanus injection. However, in case of mild burn injuries, many caregivers resorted to home remedies such as toothpaste, coconut oil, and fermented ice paste applied on the burnt skin, while others used framycetin sulfate cream (an antibiotic ointment) to prevent blister formation. Some caregivers reported that applying toothpaste on the burnt surface had a cooling and analgesic effect. When questioned about its effectiveness, some of them answered that they had a positive experience using toothpaste to reduce the burning sensation to a reasonable extent.

The caregivers used several first aid techniques for children who consumed poisonous substances such as kerosene and rat poisons, commonly induced vomiting by giving the victims saltwater and tobacco leaf to chew. Caregivers’ first-aid approaches to suffocation varied according to the type of suffocation the child experienced. A caregiver described resuscitating a child who almost drowned.

I pulled him out and made him lay upside down, then slightly hammered him by my hands on his back and made him discharge the water from his mouth. At the same time, I took off his clothes and gave him warmth with an electric heater and applied mustard oil all over. (FGD number 5)

In a situation where a button was trapped in a child’s nose, the caregiver said,

*He was rushed to the hospital. (FGD number 1)*

In another situation of suffocation, where the child had something logged in her throat, the caregiver reported that,

She was given one-two back blows. (FGD number 4)

Some caregivers’ approaches to snake and scorpion bites included spiritual cleansing after the region of the bite had been tied to prevent the circulation of the toxin to the other parts of the body. However, others believe that the victims should be taken to the hospital for treatment.

## Discussion

This qualitative study discovered several themes on caregivers' perceptions and experiences of common causes, consequences, prevention, and treatments of child injuries. Similar to previous studies (Galal et al., 2016; Parmeswaran et al., 2017), the common injuries reported by caregivers in the current study were falls, drowning, suffocation, poisons, burn, and road traffic injury. Fall were identified as the most frequent childhood injury, and this finding is in line with previous studies (Chaudhary et al., 2018). In addition, participants also reported other causes of child injury, including injuries occurring due to metallic objects (including electric shock) and injuries caused by animals (including dog bites, snake bites, scorpion stings, and chase by buffalo, cows, and monkeys). The data showed that the caregivers generally had good knowledge of common childhood injuries.

Child injury is of global health importance as it is a significant cause of disabilities (Sleet, 2018). Non-fatal injuries, as reported by caregivers, have resulted in varying degrees of consequences, including emotional distress, pains, infections, scar formation, physical disability, phobia, loss of eyesight, head injury, and paralysis. The stigma attached to child injury is often long-lived and may remain with the children for the rest of their lives. For example, research has shown that victims of burn injury and their siblings experienced stigma (Lehna, 2013). As mentioned in the group discussions, the parents of children who developed disabilities, including blindness secondary to injuries and scars from burns, experienced emotional stress. This finding is in line with previous research highlighting the effect of child injury on parental mental health (Meijel et al., 2020).

On the whole, most caregivers blamed the children for their injuries rather than owning responsibility for their occurrences or even understanding that the unsafe physical environment surrounding children might be at fault. They emphasized that children were mischievous, playful, and thus responsible for their injuries throughout the conversations. Another opinion expressed by the caregivers was fatalism about injuries such as falls, thus indirectly reflecting their acceptance of preventable injuries. Previous studies have reported similar caregiver perceptions outside India (Bennet Murphy, 2001).

Most caregivers reported using few environmental modification strategies, such as restricting children’s access to potential hazards. However, they failed to realize and acknowledge that many of the injuries, for example, burns caused by hot cook stoves placed on kitchen floors, can be attributed to the lack of a safe child-appropriate physical environment and thus can be prevented through environmental modification. Previous research has reiterated the pivotal role of the surrounding physical environment in facilitating or preventing unintentional child injury (Ablewhite et al., 2015; He et al., 2014). Therefore, interventions aimed at changing the attitude of caregivers regarding the importance of the environment can be beneficial in child injury prevention in this setting. Furthermore, a few of these hazardous physical environmental attributes such as cooking on the floor, children playing with polythene bags instead of child-appropriate playthings might reflect the low-socioeconomic status of the household. This finding is in line with previous research that has provided evidence connecting the risk (Yuma-Guerrero et al., 2018) and outcome (Kruithof et al., 2017) of unintentional child injury to socioeconomic deprivation of the family. However, most of this data is from developed countries. Therefore, the scope of extrapolating those conclusions to resource-limited settings like India is limited.

Improved caregiver supervision can prevent many of the described injuries. Adequate supervision of children is essential because adults influence children to change their risk-taking behaviour and might be effective in minimizing the potential adverse effect on injury risk of some child attributes such as the tendency of disruptive behaviour (Morrongiello & Schell, 2009). Some caregivers in FGDs were aware of this fact and believed that proper supervision of their children, whether at home or in schools, is an effective method of preventing injuries. Caregivers also feel that injury prevention should be taught at schools because children trust whatever their teachers say. However, the research supporting this notion is inconclusive, as reported in a 2016 systematic review and meta-analysis and thus requires further study (Orton et al., 2016).

Another theme that emerged from these discussions was that caregivers reported using first-aid measures to save the lives of the children, which can be considered inappropriate. For instance, the application of toothpaste, coconut oil, creams, and ice on burns (Hamdiya et al., 2015) is not an effective first-aid for burns and may potentially cause more harm than good. In contrast, appropriate first-aid for burns consists of irrigating the burnt area with running tepid water to wash off any chemicals and/or to cool down the surface temperature followed by covering with sterile cling wrap till medical attention is available (Hudspith & Rayatt, 2004). However, none of the participants mentioned this in their discussion. Similarly, inducing vomiting in poison-related injuries is a highly controversial method of poisoning management, especially since people with no medical knowledge might not distinguish between corrosive and non-corrosive poisons, and induced vomiting is strictly contraindicated in corrosive poisoning (Chibishev et al., 2012). These findings are concurrent with those of a recent study in which caregivers were reported to lack appropriate first-aid knowledge to intervene when a child consumes poison (Pathak et al., 2019). Thus, these results suggest a clear need for interventions that can educate parents and caregivers on appropriate first aid measures, preferably with the distribution of proper first aid kits containing ointments and other topical agents intended for minor burns.

### Strengths and Limitations

While the current study is one of the few that focus on caregiver perceptions and experiences of childhood injury in India and presents essential information regarding the same, its findings should be interpreted in light of its limitations. First, all of the caregivers in the study were female, which means that the perspectives of male caregivers on child injury could not be obtained. Traditionally, females, especially mothers, have been considered the primary caregivers of children (Gunnarsdottir et al., 2013; Kotila et al., 2013), and this practice is true for most Indian communities (Isaac et al., 2014). However, fathers’ perspectives and experiences are also critical especially considering the increase in the active involvement of fathers in child-rearing (Sekaran et al., 2020), and thus, future research should attempt to elicit opinions of caregivers of both genders. Furthermore, the current study collected data from several villagers, thus preventing an in-depth analysis of injuries in a given setting.

## Conclusion

Overall, caregivers in these communities have experiences in different types of child injuries and their causes. They have demonstrated some knowledge of treatment and prevention of injuries. However, the results suggest that educational interventions targeted at child caregivers regarding appropriate environmental modifications, injury prevention, and immediate post-injury management are required to reduce the occurrence of preventable unintentional child injuries.

## Supplementary Information

Below is the link to the electronic supplementary material.

Supplementary file1 (PDF 396 kb)
