# Supplementary material for: Perceptions and Experiences of Caregivers on Child Injuries: A Qualitative Study from Central India
Source: J Prev (2022). 2022 May 27;43(4):549–65. doi: 10.1007/s10935-022-00682-3 (PMC9252948; doi:10.1007/s10935-022-00682-3)
Supplement: Supplementary file 1 — Supplementary file1 (PDF 396 kb) [file 10935_2022_682_MOESM1_ESM.pdf]

Supplementary Material

Title: Perceptions and experiences of caregivers on child injuries: A qualitative study from central India.

Supplementary Table 1: Example of analysis and description of themes, categories, subthemes and quotes

	Themes	Categories	Subthemes	Quote code with FGD number
1	Caregivers' perception and experiences of common causes of child injuries	Fall	Learning to walk<br>Playing<br>Climbing staircases	"*This boy is very mischievous. As he plays inside, he falls [demonstrates]. He is so naughty that, recently, he fell and hit his head on the gate." (FGD number 3)*<br><br>*"While children were playing, they bumped into one another, and she fell, resulting in injuries. On another occasion, she was sitting, and another child pushed her. She fell off the bed leading to bone injury, and she could not raise her hand properly for eight days." (FGD number 4)*
		Road Traffic Injuries	Motorcycle<br>Car crash<br>Collision with pedestrians	*"The driver suddenly applied the brake, and he hit the boy. The vehicle rider was kind enough, felt regret and carried him to our door on the upper floor; otherwise, we would not have known of the incident." (FGD number 3)*
		Metallic nails and tools injuries	Scissors<br>Stepping on nails	*"It was the gallery, where children were playing. The little kids prepared dresses for dolls, cutting the clothes with scissors. We didn't know how the scissors pierced her eye, and the black lid of the eye was severely damaged, and she lost her vision. She was a young girl of age between 8 and 10 years old." (FGD number 6)*
		Injuries caused by animals	Dog bite<br>Snakebite<br>Scorpion stings	*"A dog bit my elder daughter. Nine years have passed; still one can see the mark on her skin". (FGD number 6)*

		Burns	Hot water<br>Hot tea<br>Hot frying pan	*"I was cooking, and he was sitting near the burner. He slipped and fell on the hot frying pan. He got burnt and started bleeding. We had to take him to the hospital for treatment". (FGD number 5)*
		Ingestion of foreign objects and poisons	Coin<br><br>poisons	*"She ate fertilizer used on wheat thinking it was sugar; it is like sugar. We were all worried and confused about how much she had eaten. I was sitting inside. On our way back, suddenly, she started vomiting many times. She fainted while vomiting, and we had to rush her to the hospital". (FGD number 7)*
		Drowning	Open well/<br>Tank	*"When my son was in his childhood, he fell inside the water tank while running around and shouting lizard. He fell in the water tank, and if he had been left there for a second more, the scenario would have been different." (FGD number 4)*
		Suffocation	A trapped object inside the throat, Breastfeeding	*"There is a threat of eating bananas. A boy died at Subhash Nagar while eating a banana. He ate hastily, and it entered the respiratory tract". (FGD number 4)*
2	Consequences of child injuries,	Pains	Fall, Road traffic injuries	*"[Imagine] if a spectator can feel the pain, how much pain the injured fellow might be feeling" (FGD number 1)*
		Infection<br>Scar formation	Falls,<br>Burns	*"A dog bit my elder daughter. Nine years have passed; still one can see the mark on her skin". (FGD number 6)*
		Physical disability	Fall, Road traffic accident	*‘‘This resulted in broken legs; still, he cannot walk on his own" (FGD number 1)*
		Loss of eyesight	Injury to the eye from scissors	*"It was the gallery, where children were playing. The little kids prepared dresses for dolls, cutting the clothes with scissors. We didn't know how the scissors pierced her eye, and the black lid of the eye was severely damaged, and she lost her vision. She was a young girl of age between 8 and 10 years old." (FGD number 6)*

		Head Injury, Paralysis	Fall, Road traffic accident	*''Among our three children, one child is too weak after falling. She developed neurological disorders as per our assumption. She feels uneasy writing something. She frequently falls with her face down. For nine-month, she could not take meals because of the injury. However, she could speak out properly but could not write correctly. She writes one page in one hour. Dr Bhoraskar had declared her mentally challenged.'' (FGD number 8)*
		Phobia, Emotional Stress	Burns Blindness	*''Since he was burnt, he developed a phobia. He is too much scared of being left behind in a closed room. He runs out there. He is scared when the fan is working. You have to put on the light. He is scared of television and high-pitched sound''. (FGD number_)*
		Death	Fall, road traffic accident, burns, drowning and suffocation	*''A manual worker gave birth nearby Diwali. She cared for the baby for a month, and one day, as she was feeding the baby, she slept off and continued feeding the baby lying down. Within ten minutes, the baby died.'' (FGD number_)*<br><br>*''The boy, three years old, was playing with a balloon. While blowing the balloon, it stuck in his throat, and he died on the spot''! (FGD number_)*
3	Caregivers' experience of first aid treatment during injury	Pressure application	Fall	*''We did not do much; we covered and pressed the head with a scarf smoothly''. (FGD number1)*
		Paste-on burns	burns	*''if we apply toothpaste, the victim will feel relieved'', while another caregiver said, ''if we apply wet sludge, it controls the burn itching effect''*. However, another caregiver said, *''we will immediately rush the victim to a hospital in case of serious burn''. ''Soframycin cream was applied and with fomented with ice.'' (FGD number_)*

		Induced vomiting	poisons	"we would dissolve ample amount of salt in water and make him drink the same and give him tobacco to chew; the tobacco will compel him to vomit." (FGD number_)
		Spiritual cleansing	Snakebite, scorpion stings	"granddad had faith in mother divine, and we prayed to mother idol Inderkheda, and she regained her senses after a while and got better in due course." (FGD number_)
		Resuscitation	Drowning	"I pulled him out and made him lay upside down, then slightly hammered him by my hands on his back and made him discharge the water from his mouth. At the same time, I took off his clothes and gave him warmth with an electric heater and applied mustard oil all over." (FGD number_)
4	Caregivers' experiences of Injury prevention	Keeping medicines in safe places	poisons	"We keep them away from the children" and "we do not allow them to hold it." (FGD number_)
		Keeping children indoor	All categories	"We retain them at home, allowing them to watch television." (FGD number_)
		Injury prevention education in schools	All categories	"We decided to admit the little kid at playschool to prevent them from going out and getting injured by animals." (FGD number_)
		Constant supervision		"We have to keep attention everywhere".(FGD number_)

Appendix 1

open-ended interview guide

<u>Proposed Topic Guide for Parents/ Guardians</u>

1. **What are common childhood injuries that you see/encounter at your home /village /locality**

   Probe: What type, common places, Physical injuries, poisoning, burn, injuries (infant, child, adolescent), different reasons for childhood injuries.

2. **Can you share some personal experiences regarding childhood injury in your family, locality and village**
   Probe what happen, where, what you did first, severity, health care

3. **What are the long term and short term consequences of childhood injury**

4. **What are common physical structures in your house, locality, villages that you think can lead to a childhood injury?**

5. **Can you elaborate burn injuries, its causes and how to prevent those injuries**

6. **Can you elaborate on poisoning and agricultural related, its causes and its prevention**

7. **Can you elaborate on drowning, its causes and its prevention**

8. **Can you elaborate on suffocation, its causes and its prevention**
   Probe: One child itself, on Family and on Society, economic, social impact etc

9. **Can you give some suggestion how to prevent childhood injuries at home and in your village**
10. **What approaches /method you apply to educate your children to prevent injuries amongst them**
